# Genetically Distinct *Glossina fuscipes fuscipes* Populations in the Lake Kyoga Region of Uganda and Its Relevance for Human African Trypanosomiasis

**DOI:** 10.1155/2013/614721

**Published:** 2013-10-02

**Authors:** Richard Echodu, Mark Sistrom, Chaz Hyseni, John Enyaru, Loyce Okedi, Serap Aksoy, Adalgisa Caccone

**Affiliations:** ^1^Faculty of Science, Gulu University, Loroo Division, Gulu Municpality, Gulu, Uganda; ^2^Department of Ecology and Evolutionary Biology, Yale University, 21 Sachem Street, New Haven, CT 06520, USA; ^3^School of Biological Sciences, Makerere University, Kampala University Rd, Kampala, Uganda; ^4^National Livestock Resources Research Institute, P.O. Box 96, Old Busia Road, Tororo, Uganda; ^5^Department of Epidemiology of Microbial Diseases, Yale School of Public Health, New Haven, CT 06520, USA

## Abstract

Tsetse flies (*Glossina* spp.) are the sole vectors of *Trypanosoma brucei*—the agent of human (HAT) and animal (AAT) trypanosomiasis. *Glossina fuscipes fuscipes* (*Gff*) is the main vector species in Uganda—the only country where the two forms of HAT disease (*rhodesiense and gambiense*) occur, with *gambiense* limited to the northwest. *Gff* populations cluster in three genetically distinct groups in northern, southern, and western Uganda, respectively, with a contact zone present in central Uganda. Understanding the dynamics of this contact zone is epidemiologically important as the merger of the two diseases is a major health concern. We used mitochondrial and microsatellite DNA data from *Gff* samples in the contact zone to understand its spatial extent and temporal stability. We show that this zone is relatively narrow, extending through central Uganda along major rivers with south to north introgression but displaying no sex-biased dispersal. Lack of obvious vicariant barriers suggests that either environmental conditions or reciprocal competitive exclusion could explain the patterns of genetic differentiation observed. Lack of admixture between northern and southern populations may prevent the sympatry of the two forms of HAT disease, although continued control efforts are needed to prevent the recolonization of tsetse-free regions by neighboring populations.

## 1. Introduction

Tsetse flies, *Glossina* spp. (Diptera: Glossinidae), are the sole vectors of the trypanosomes causing human (HAT) and animal (AAT) African trypanosomiasis [1]. Currently, there are no vaccines, and available drugs are expensive, toxic, and logistically difficult to administer [2, 3]. As vector density reduction can effectively reduce HAT transmission [4, 5], in 2001, the African Union established the Pan African Tsetse and Trypanosomiasis Eradication Campaign (PATTEC) for a large-scale control of HAT and AAT [6]. Control methods used in the campaign include sterile insect technique (SIT), odor-baited insecticide-treated traps, live baits, targeted aerial insecticide spraying, and sequential aerosol [7–13].

The efficacy of the methods that target vector population reduction can be improved by understanding the population dynamics of the vector species. Genetic tools can be very helpful in this regard, as they allow for identifying barriers to gene flow, predicting fly movements, and assessing reinvasion risks from neighboring sites, where control is not implemented [14]. Population genetic studies can also define the spatial extent, temporal stability, and size of the population targeted for control and thus help determine the appropriate scale at which control can be effective. This information has become a vital tool in guiding the implementation of tsetse control strategies geared towards suppression and complete elimination of flies [15], since the pattern of spatial genetic structure provides quantitative information on population densities and dispersal rates, which are important parameters for designing an efficient control strategy [16]. For example, the density of traps or targets impregnated with insecticides needed to reduce tsetse densities will depend on the dispersal capacities of the flies [17]. The number of sterile males and the distance between release sites to achieve an SIT campaign will also depend on the abundance and dispersal capacities [18–20].

Population genetic studies of riverine *G. palpalis gambiensis* in Guinea and Senegal have identified populations that are sufficiently isolated to warrant attempts at complete eradication [15, 21]. Population genetic studies in *G. p. gambiensis* and *G. p. palpalis* in Senegal, Burkina Faso, and Equatorial Guinea show patterns of high gene flow characterized by spatial and temporal heterogeneity influenced by landscape fragmentation [15, 22, 23]. These studies demonstrate the importance of gene flow in determining the degree of fine-scale genetic structure, the size of the local genetic neighborhoods within populations [15, 23], and the need to integrate information regarding barriers to gene flow in tsetse elimination schemes [14].

In Uganda, *Gff*, a riverine subspecies in the *palpalis* group, is the major HAT transmission vector. Except for a disjunct region in Ethiopia/Sudan, Uganda and western parts of Kenya represent the eastern edge of its range [28], where it occurs in localized vegetation thickets along water bodies, which offer tsetse seasonal refugia and access to host species in their search for food and relief from heat. *Gff *densities are strongly influenced by ecological and climatic features, since temperature and precipitation may change the vegetation landscape and thus density and size of tsetse populations [29–31].

 Ecological data suggest that *Gff* has great capacity for dispersal and recolonization of suitable habitats [32]. Such rapid dispersal within the habitat would cause genes to spread rapidly leading to genetic homogenization of tsetse flies across the geographic landscape. This is not what genetic studies from our group have revealed. The genetic screening of about 37 *Gff *populations across Uganda showed that this taxon is structured into three major genetic population clusters, with a southern and northern cluster separated by Lake Kyoga [24] and a third one present in western Uganda [25]. Although gene flow can occur between these genetic clusters with a few migrants detected over a radius of about 100 km, genetic mixing is quite frequent between the northern and southern population genetic clusters along a contact zone along Lake Kyoga in the areas of Bunghazi in eastern, Masindi in western, and Junda in central Uganda. Our studies have also suggested female-biased dispersal into the contact zone from sites within the southern cluster [25]. Genetic studies also demonstrated the temporal stability of *Gff *populations in Uganda, including those from the contact zone [20, 26, 33].

The patterns of genetic differentiation between *Gff* populations might also be impacted by the symbiotic bacteria they carry. All tsetse species harbor a vertically transmitted mutualistic symbiont, *Wigglesworthia glossinidia*, which is necessary for host physiology. As expected, the genetic structure of *Wigglesworthia *reflected the *Gff* host mtDNA patterns of genetic differentiation [34]. On the other hand this congruence between host mtDNA and parasite patterns of genetic diversity was not found in another maternally inherited bacteria, the parasitic *Wolbachia *[34–36]. This symbiont has been shown to manipulate host reproduction in *Glossina morsitans morsitans, *causing cytoplasmic incompatibility (CI) in the laboratory; crosses between *Wolbachia* infected females and uninfected males result in embryonic lethality, while the reciprocal cross are fertile [37]. Our studies on *Wolbachia* in *Gff* in Uganda has shown that infection prevalence and density in different populations vary, and that individual flies can carry more than one *Wolbachia* strain [34]. Thus, *Wolbachia* mediated incompatibilities between populations can contribute to the genetic disjunction we observe in *Gff* as a result of CI mediated effects.

Due to lack of obvious vicariant barriers, the contact zone was speculated to result from secondary contact of flies following allopatric divergence and expansion [25]. However, the limited sampling of the region did not allow for the determination of its precise geographic extent and dynamics. The low vagility of *Gff* together with its tendency to cluster in discrete habitats (thickets of vegetation along river bodies) and its strong association with density-independent factors suggest that local adaptation to environmental parameters may also contribute to the maintenance of population divergence. This implies that habitat availability will largely control densities of populations and their connectivity. 

The *Gff* populations in northwestern Uganda transmit only the *gambiense* form of HAT caused by *Trypanosoma brucei gambiense*, while *Gff* populations in the southern genetic clusters transmit the *rhodesiense* form of HAT caused by *T. b. rhodesiense*. In recent years, the *rhodesiense* disease has been expanding its range from the historical loci in the southeast into new foci in central Uganda north of Lake Kyoga [35]. In fact, the two disease belts are separated only by a disease free belt of less than <100 km just north of Lake Kyoga [37]. Since the pathology, diagnosis, and treatment vary between the two forms of disease, a potential merger of the two disease belts would cause major public health crises [2]. Thus, understanding the vector dynamics in this contact zone will provide insights on the potential risk of the sympatry of these two currently allopatric HAT forms, which were never in contact before. If the flies can acquire and transmit both forms of the human parasite, this could result in unknown epidemiological outcomes since the parasites can undergo recombination in the tsetse salivary glands [20, 38–42].

In the current study, we comprehensively sampled the area where the genetically differentiated populations of *Gff *are in contact in central Uganda and used multilocus genetic data to examine population structure and dynamics and to evaluate if environmental differences might be involved in maintaining the genetic difference between the two genetic clusters north and south of Lake Kyoga. We discuss our data on the stability of the different genetic populations around Lake Kyoga in the context of the potential merger of the two disease belts and the ongoing vector control programs.

## 2. Materials and Methods

### 2.1. Tsetse Collection and Study Area

**Figure 1:**
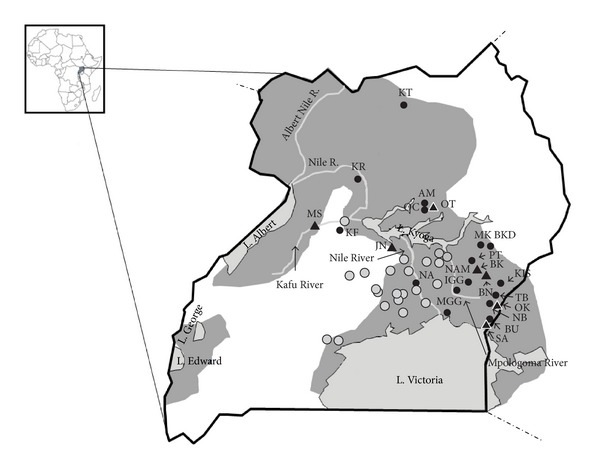
Localities where *Glossina f. fuscipes* samples were collected. The inset map shows the geographical position of Uganda in Africa. The dark grey shows the current species range. Lakes are indicated by name and with a light shade of grey. The three major rivers (Nile, Kafu, and Mpologoma) are also indicated by name. Sampling sites are identified by abbreviations and expanded in Table 1 and Supplementary Table  S1 (see Supplementary Material available online at http://dx.doi.org/10.1155/2013/614721). Empty circle corresponds to areas previously sampled in Abila et al. [24], Beadell et al. [25], and Echodu et al. [26]. Circles indicate new sampling sites (black circles: new sites with tsetse; grey circles: sites with no tsetse) and triangles indicate sites examined in previous studies.

Tsetse flies were collected using biconical traps [43] during field expeditions between 2009 and 2012 from 49 sites. Sampling details are summarized in Figure 1, Table 1 and Supplementary Material. Collections in each locality were carried out for 3 days with an average of 6 traps per site. Traps were located within a radius of 5 km^2^. Each fly was stored individually in 90% ethanol. Localities generally reflect the riverine/woodland habitat preferred by *Gff*.

### 2.2. Genetic Data Collection

DNA was extracted from tsetse legs using PrepGEM Insect DNA extraction kit (ZYGEM 79, New Zealand) or DNeasy kits (Qiagen, USA). PCR was used to amplify a 530 base pair (bp) fragment of the mitochondrial gene cytochrome C oxidase II (COII) from a subset of flies from each locality following Beadell et al. [25]. Individual flies were genotyped at 18 microsatellite loci (Supplementary Material) using previously described protocols [25, 44, 45] with the exception of C07 and GmL11, where 0.5 units of Taq Gold polymerase (Life Technologies, USA) were used. PCR products were multiplexed in groups of two or three loci and then genotyped on an ABI 3730xL sequencer (Life technologies, USA). Alleles were scored using Genemarker v2.4.0 (SoftGenetics, USA) with manual editing of the automatically scored peaks. 

### 2.3. Statistical Analyses of Genetic Data

We tested microsatellite loci for within site deviations from Hardy-Weinberg equilibrium (HWE) and linkage disequilibrium (LD) using Genepop v4.0 [46]. Markov chain parameters were set at 10,000 dememorizations, 1000 batches, and 10,000 iterations per batch. Locus and locality specific estimates of microsatellite allele frequencies were generated using GenAlex v6.4 [47]. We used the Fstat v2.9.3.2 [48] to calculate site specific inbreeding coefficients (Fis) and Arlequin v3.5 [49] to calculate allelic richness (*A*) and observed (*H*_*O*_) and expected (*H*_*E*_) heterozygosity of populations. DnaSP v5.0 [50] was used to calculate mtDNA haplotype (Hd) and nucleotide (*π*) diversity.

For all sites from which we had temporal samplings, we characterized the proportion of the variance attributable to differences in sampling dates using the analysis of molecular variance (AMOVA) as implemented in Arlequin v3.5 [49] on both microsatellite and mtDNA datasets. For the microsatellite dataset we calculated pairwise *F*_ST_ using Genepop v4.0 [46] and generated locus and population specific estimates of microsatellite allele frequencies using GenAlex v6.4 [47] for the different temporal samplings. Temporal samples that were not genetically distinct were pooled in subsequent analyses.

Overall genetic differentiation among localities was assessed by estimating pairwise *F*_ST_ values [51], and significance was determined using Fisher's G-based exact test for genotypic differentiation using Genepop v4.0 [46]. For both microsatellite and mtDNA data, we used an analysis of molecular variance (AMOVA), in Arlequin v3.1 [49], to analyze the partitioning of genetic variance within and between sampling localities. Relationships among haplotype lineages were inferred by constructing a parsimony network using TCS v1.21 [52].

Population structure was inferred from microsatellite data using the Bayesian clustering method implemented in STRUCTURE 2.3 [53]. Three independent runs for each *K* = 1–15 were carried out. For all runs, an admixture model and independent allele frequencies were used with a burn-in value of 250,000 steps followed by 1,000,000 iterations. The optimal value of *K* was determined using STRUCTURE HARVESTER v0.6 [54] to calculate *ad hoc* statistic “Δ*K*” [55].

To determine whether the patterns of genetic structure were a result of sampling related individuals, we estimated relatedness and examined relationships for all individual pairs using the program ML-Relate [27]. Each study site was run individually to determine if sites differed significantly in the proportion of related individuals detected. We compared results for individuals from which we had both mtDNA and microsatellite data to identify mismatches between data types ((10) of the Appendix). 

To identify first generation migrants, we used the Bayesian approaches implemented in STRUCTURE and GeneClass 2.0 [53, 56]. Previously obtained STRUCTURE results were used to assign individuals to each of the *K* populations. Samples were placed into the respective population based upon the highest percentage of membership (*q*) using a threshold value of *q* ≥ 0.90 [57]. We used the “detect migrants” function of GeneClass to calculate the likelihood of finding an individual in the locality in which it was sampled (*L*_*h*_), the greatest likelihood among all sampled localities (*L*_max⁡_), and their ratio (*L*_*h*_/*L*_max⁡_). Because migrants from unsampled populations can be misclassified as residents, we selected the Rannala and Mountain [58] criterion with the resampling method of Paetkau et al. [57] to determine the critical value of the test statistics, *L*_*h*_ and *L*_*h*_/*L*_max⁡_, using 1,000 simulated individuals and the default 0.01 Type I error (*α*).

We used Fstat v2.9.3.2 [48] to test whether the observed population structure could be attributed to differences in dispersal between sexes and used four statistics: differentiation among populations (*F*_ST_), mean assignment indices (mAIc), the variance in assignment indices (vAIc), and mean pairwise relatedness (mPr) [48, 59, 60]. 

## 3. Results 

### 3.1. Sampling

 We visited 49 sites spanning the known *Gff* distribution but could trap flies in only 23 sites despite similar environmental conditions and equivalent collection efforts. We collected 2918 tsetse flies with an average of 127 per locality (Figure 1 and Table  S1). The absence of flies or the extremely low (*n* < 2) capture rates occurred at sites near cattle corridors, where farmers apply synthetic pyrethroid acaricides for tick and tsetse control on cattle weekly, and in districts subjected to control efforts for tsetse flies using insecticide treated traps [9]. In addition, low capture rates of flies in a trap do not necessarily reflect the abundance of tsetse in an area, as trap efficiencies for *Gff* are particularly low [60].

### 3.2. Genetic Diversity

In all analyses for both microsatellite and mtDNA markers, we included published data from seven sites [25, 26]. The final data set for the microsatellite loci analyses included 23 sites and 1221 flies averaging 53 flies/site. For the mtDNA analyses, we screened a subset of these samples (244 flies from 19 sites, averaging 13 flies/site).

Of the 18 microsatellite loci, GpC29 and C5 did not adhere to HWE expectations (Supplementary Material) and were excluded from further analysis. Pgp17 was also excluded because of scoring inaccuracies likely due to the large range of allele sizes (70 bp to >200 bp). We detected no significant linkage among the 15 remaining loci. Overall, all sites showed moderate to high levels of genetic variability; *A*_*R*_ ranged from 2.900 to 7.677, *H*_*O*_ ranged from 0.487 to 0.604, *H*_*E*_ ranged from 0.46 to 0.652, and FIS ranged from −0.015 to 0.126 (Table 1). *H*_*E*_ and *H*_*O*_ microsatellite diversities were similar, indicating random mating within sites. 

**Figure 2:**
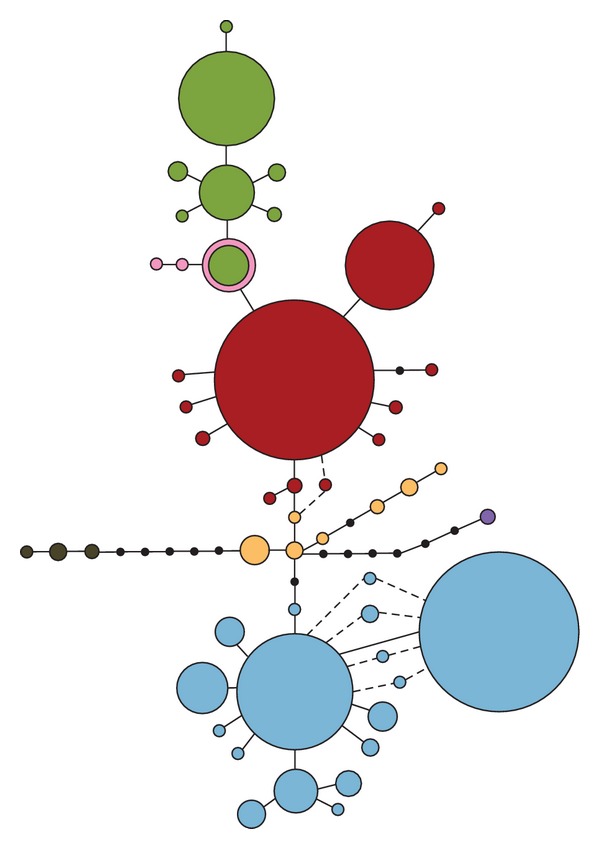
Parsimony network of mitochondrial haplotypes recovered from *G*. *f*. *fuscipes* and their distribution in Uganda, Kenya, and Sudan. Haplotypes are represented by circles. Their size is proportional to their frequency. Haplotypes are shaded to represent genetic populations—Green is western, red is northern, blue is southern, and yellow is intermediate. The purple haplotype represents a divergent haplotype discovered in MGG, pink haplotypes are from a disjunct population of *G*. *f*. *fuscipes* in Sudan, and brown haplotypes are from a population of *G*. *f*. *quanzensis* from the Democratic Republic of the Congo.

 The mtDNA dataset consisted of 489 COII sequences (530 bp), including 244 new and 245 published sequences [25, 26], and resulted in a total of 57 haplotypes, including 15 new ones (Figure 2(a), Supplementary material). The number of haplotypes at each site ranged from 1 to 4, haplotype diversity (Hd) ranged from 0 to 0.714, and nucleotide diversity (*π*) ranged from 0 to 0.00664 (Table 1). In 12% of the samples (26 individuals) cluster assignment for mtDNA and microsatellite data was incongruent ((10) of the Appendix).

### 3.3. Temporal Variation in Genetic Diversity

We tested for differences in genetic diversity between samples collected in 2008 and 2011 at 4 sites ((5) of the Appendix). AMOVA using both microsatellite allele frequencies and mtDNA haplotype frequencies suggested that differences between temporally-spaced samples were not significant; however, differences among localities were significant (Supplementary Material). The variation explained by site was greater for mtDNA than for microsatellite data. Pairwise *F*_ST_ values between samples were also low (Table  S6). MtDNA haplotype frequencies were not different in three of the four sites between the two time points for each site (MS, KF, or KR, Figure  S1), although some change was observed in BN, likely due to the loss of the two least common haplotypes in the 2011 samples.

### 3.4. mtDNA Network Analyses

**Figure 3:**
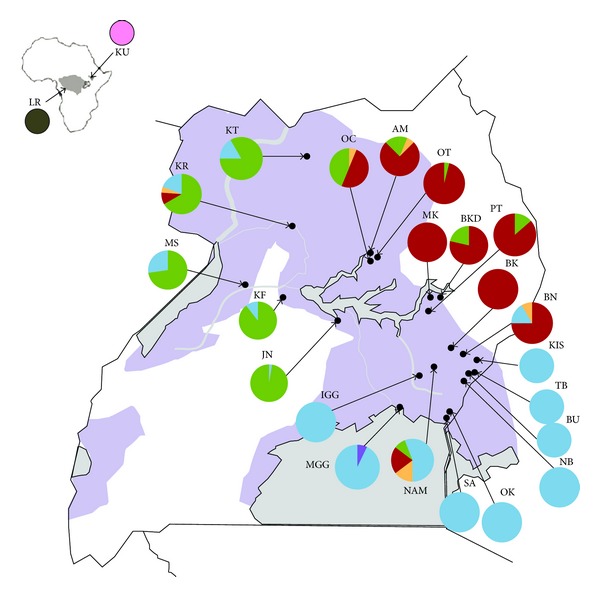
Geographic distribution of mitochondrial sequence diversity. Pie charts on maps indicate the frequency with which mtDNA haplotypes (identified in Figure 2) occurred in each sampling site. Background shading indicates the predicted range of *G*. *f*. *fuscipes* in the region and in Africa (inset). Haplotypes are shaded to represent genetic populations—Green is western, red is northern, blue is southern, and yellow is intermediate. The purple haplotype represents a divergent haplotype discovered in MGG, pink haplotypes are from a disjunct population of *G*. *f*. *fuscipes* in Sudan, and brown haplotypes are from a population of *G*. *f*. *quanzensis* from the Democratic Republic of the Congo. The dark grey shows the current species range.

 The phylogenetic relationships among the 57 mtDNA haplotypes are shown in Figure 2 and confirm the existence of the two major northern (N) and southern (S) haplogroups [25] with most of the new haplotypes included in one of the two haplogroups. The increased sampling density allowed for the recovery of intermediate haplotypes between the two major haplogroups, and also of more distantly related haplotypes, separated by a maximum of 10 substitutions from the most common haplotypes from each haplogroup. The haplotype geographic distribution is shown in Figure 3; both N and S haplogroups were found in 6 sites (KF, KR, MS, JN, NAM, and BN), 3 of which (BN, JN, and MS) had been previously identified as having both haplogroups. 

### 3.5. Measures of Genetic Differentiation and Structure

**Figure 4:**
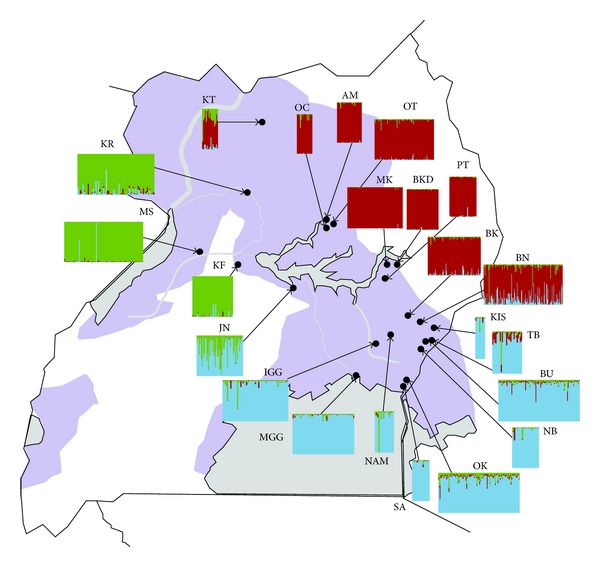
Geographic distribution of the three genetic clusters detected using microsatellite data based on STRUCTURE results. Each rectangle depicts the genetic assignment of each individual from that sampling site. Each individual within each rectangles is represented by a vertical bar, with assignment to the three genetic clusters shown as the proportion of each color making up each bar. Colors represent genetic populations—Green is western, red is northern, and blue is southern. The dark grey shows the current species range.

 Bayesian clustering analyses performed in STRUCTURE identified 3 genetic clusters including multiple sampling sites—northern (KT, AM, OC, OT, MK, BKD, PT, BK, and BN), southern (NAM, IGG, MGG, KIS, TB, BU, NB, OK, and SA), and western (KF, MS, and KR). The analysis also showed admixture across genetic clusters with flies from Junda (JN) showing significant ancestry with flies from both southern and western genetic clusters (Figure 4). 

*F*_ST_ values between sites based on microsatellites ranged from 0.06 to 0. (Supplementary Material). *F*_ST_ values between clusters detected by STRUCTURE ranged from 0.14 to 0.21 (Supplementary Material). AMOVA results using microsatellite allele frequencies (Table 2) showed that differences both within (79.3%, *P* ≥ 0.01) and between (18.03%, *P* ≥ 0.01) sites were significant, though most of the variation was between individuals within sites.

### 3.6. Relatedness, Dispersal and Migration

Measures of relatedness based on microsatellite variation using both likelihood and Bayesian methods showed that the majority (85.8%) of the individuals are unrelated (Table 3). Migrant detection using GeneClass and STRUCTURE yielded similar results (Table 4 and Supplementary Material). STRUCTURE results indicated that although there was significant genetic differentiation between clusters, there was also gene flow between them, as 69 migrants (33 males and 36 females, Supplementary Material) were identified. While most migrants moved between sites within the genetic clusters, a small number moved between them. Within the northern cluster our analyses identified 5 migrants: one female with southern ancestry, one male with ancestry in the mixed southern/western locality (JN), and two females and one male with ancestry to the western cluster. Within the southern cluster we detected 3 migrants: two males with ancestry in the mixed southern/western locality (JN) and one male with ancestry to the western cluster. In the western cluster we found 2 migrants: one male individual with southern ancestry and one male individual with ancestry in the mixed southern/western locality (JN). In this site we inferred 7 migrants: six females and one male all with southern ancestry. 

The four statistical methods implemented did not detect significant differences in sex-biased dispersal in both one-sided and two-sided tests (Table 5). 

## 4. Discussion

In this study, we undertook an expanded temporal and spatial analysis of population genetic structures of *Gff* flies collected in central Uganda where multiple genotypes from genetically distinct population clusters were shown to exist in a zone of contact. Our analyses included multiple sampling sites and identified three main genetic clusters with northern, southern, and western distributions in concordance with previous studies [25, 26]. We find a narrow contact zone between northern and southern genetic clusters with low levels of migration between clusters. 

Increased sampling in the Lake Kyoga region revealed a much higher spatial resolution of the contact between these three clusters and the associated admixture zone. However, our additional mtDNA sampling recovered intermediate haplotypes between the northern and southern haplogroups, shown in yellow in Figure 2, reducing the division between these two populations groups and suggesting that their separation was more recent than previously thought [25]. Additionally, mtDNA analysis revealed many more sites at which northern and southern haplotypes occur sympatrically (Figure 3) than previously detected, providing greater spatial resolution of the contact zone. The contact zone between the three clusters appears to be relatively narrow, extending through central Uganda to the western areas in Karuma and Kafu. All admixed sampling localities are along the path of major rivers; the Mpologoma River, the Kafu River, and the Nile River that drain into and out of Lake Kyoga (Figure 1). The narrow extent of this contact zone, in addition to the apparent south to north direction of mitochondrial introgression between the northern and southern population clusters, supports the role of contiguous riverine habitat facilitating the dispersal of tsetse flies along watercourses [14, 25]. Despite relatively short distances between sites within different genetic clusters (e.g., 10.5 km between NB and OK), we detected significant genetic differentiation between them (Supplementary Material), which is concordant with previous studies [25, 26, 61].

There was a substantial mismatch in the assignment of mitochondrial haploytpes and ancestry based on microsatellite data. This mismatch could be explained by sex bias in dispersal, as has been detected in other population studies of *Gff* [24, 25], but we did not detect any significant bias in this study. It is also possible that *Wolbachia*-induced mating incompatibility is driving the differentiation between the population structure inferred from mitochondrial and nuclear DNA. *Wolbachia *mediated effects on host fitness and host population genetic can drive patterns of mtDNA variation regardless of the nuclear DNA background [36, 61, 62]. A finer scale analysis of *Wolbachia* infection prevalence and strain types present in flies in the contact zone is necessary to further clarify the role of *Wolbachia* mediated reproductive effects.

Migration of flies between sites from different genetic clusters is extremely low, particularly in relation to north-south migration. We detected only a single migrant from a southern locality to a northern locality (BN to NAM) and no migrants in the other direction. Despite this, it does appear that mitochondrial introgression is occurring in a south to north direction (Figure 2(b)), especially along the path of major rivers. Conversely, admixture between both northern and southern population clusters with the western one was slightly higher, particularly in the mixed JN locality. 

The contrast between the occurrence of intracluster migration and the low level of interacluster migration could be caused by two mechanisms—restriction of movement between clusters due to vicariant barriers or through reciprocal competitive exclusion of flies originating from adjacent clusters following local adaptation. As the contact zone lies around Lake Kyoga and major rivers and flies can cross riverine barriers separating sites within clusters, there is a lack of obvious physical barriers to gene flow that would explain the observed genetic differentiation among the genetic clusters seen in this study. 

Competitive exclusion could also be driving the observed pattern, and experimental mating studies could test this hypothesis. This is a compelling biological hypothesis and one that has significant implications for vector control, thus representing an important direction for future study. Environmental barriers could also be important in maintaining the genetic distinctiveness between clusters, and we have preliminary data that show a substantial gradient in climatic conditions between *Gff* sites, north and south of Lake Kyoga, suggesting congruence between the genetic differentiation of *Gff *and the differentiation of its environment which we are exploring further. 

### 4.1. Implications for *Gff* Vector Control and Future Directions

The observation of high gene flow amongst localities within the three genetic clusters indicates that control efforts, undertaken solely at local scales, are unlikely to produce long-lasting results due to reinvasion from adjacent areas and that, in the absence of continued control, areas presently free of tsetse, such as the ones in south central Uganda where control is currently enforced and that yielded low tsetse captures, are likely to become recolonized, especially given the habitat suitability of many of these regions. 

Whilst the low genetic admixture between northern and southern populations of *Gff* could suggest that the two regions they occupy could be managed as separate entities, this interpretation bears caution, as elimination of one population could result in rapid population expansion of the other. Experimental control with strict monitoring at sites in the contact zone could assist in understanding how the dynamics between these two populations change in response to control measures. In addition, this study highlights the importance of ongoing monitoring of *Gff* in Uganda to provide quantitative information on population densities and dispersal rates to inform efficient control strategies into the future.

 Future work will be directed at exploring further the underlying causes of the genetic differentiation between the three genetic clusters by performing mating experiments to look at mating compatibility, exploring further the role of *Wolbachia* mediated bidirectional CI in determining such patterns, and looking at the association between genetic and environmental variables and the impact of climatic change in shaping the distribution of *Gff *and thus disease risk.

## Supplementary Material

Appendix 1: Information on the study site, time of collection and number of flies caught in each site.
Appendix 2: Details of microsatellite markers used in the study.
Appendix 3: Per locus estimates of FIS at 18 microsatellite loci for each sampling locality.
Appendix 4: Mitochondrial haplotype information, including frequencies observed across studied populations. New haplotypes recovered from this study are indicated by ∗.
Appendix 5: Results of an AMOVA testing for temporal genetic structure in four populations of G. fuscipes sampled in 2008 and also in 2011.
Appendix 6: Fst values for temporal samples calculated on microsatellite data. Non-significant values are in bold.
Appendix 7: Microsatellite-based FST values pairwise comparison between sampling localities of G. f. fuscipes in Uganda.
Appendix 8: Microsatellite-based FST values for pairwise comparisons among the three populations detected using Bayesian clustering.
Appendix 9: Details of all first generation migrants detected by Geneclass 2.0, using Lh, Lh/Lmax and STRUCTURE.
Appendix 10: Comparison of mtDNA clade and microsatellite assignment for each individual where both data types were collected.
Figure 1: Microsatellite allele frequencies observed in seven populations of G. f. 52 fuscipessampled at different time points. Numbers after location code indicate the time interval 53 (in generations) since the first sampling.

## Figures and Tables

**Table 1:** Sample sizes and genetic diversity statistics for the mitochondrial CO1 and 15 microsatellite loci in 23 populations of *G. f. fuscipes,* on samples analyzed for this study (a) and from previous studies (Abila et al., 2008 [24]; Beadell et al., 2010 [25]; and Echodu et al., 2011 [26]).

Population	Code	Date of sampling	Microsatellites					Mitochondrial DNA			
			*N*	*A*_*R*_	*H*_*O*_	*H*_*E*_	*F*_IS_	*N*	No. haplotypes	Hd	*π*
Kafu	KF-0	Feb 2010	30	4.500	0.501	0.526	0.065	16	2	0.233	0.00345
	KF-10	Jul 2011	4	3.438	0.509	0.525	0.197	2	1	0.000	0.0000
Karuma	KR-0	Feb 2010	40	7.667	0.571	0.652	0.137	12	3	0.439	0.00641
	KR-10	Jul 2011	20	6.056	0.604	0.639	0.08	8	3	0.714	0.00664
Putiputi	PT	Oct 2009	26	4.000	0.525	0.564	0.038	22	2	0.247	0.00104
Tororo	TR	Oct 2009	2	1.944	0.472	0.347	−0.03	—	—	—	—
Iganga	IGG	Feb 2011	65	6.000	0.535	0.556	0.033	10	1	0	0
Kisoko	KIS	Feb 2011	8	3.555	0.527	0.542	−0.06	—	—	—	—
Mayuge	MGG	Feb 2011	59	5.600	0.545	0.557	0.027	15	4	0.371	0.00334
Namutumba	NAM		16	5.267	0.553	0.610	0.09	31	4	0.574	0.00517
Nambogo	NB	Jun 2011	17	4.333	0.592	0.584	−0.03	14	2	0.143	0.00272
Sangalo	SA	Dec 2011	15	4.000	0.450	0.496	0.087	15	3	0.257	0.0056
Tuba	TB	Jun 2011	28	5.267	0.579	0.583	0.005	15	2	0.248	0.00105
Aminakwach	AM	Jul 2011	30	4.733	0.537	0.555	0.068	16	2	0.125	0.00056
Bukedea	BKD	May 2011	25	4.429	0.495	0.541	0.098	14	3	0.473	0.00170
Oculoi	OC	Jul 2011	20	4.786	0.639	0.612	−0.015	16	3	0.592	0.0036
Kitgum	KT	Jan 2012	17	5.333	0.55201	0.62790	0.126	10	3	0.600	0.00184
Budaka	BK*		80	4.778	0.509	0.527	0.041	11	1	0.000	0.0000
Bunghazi	BN-0*	Mar 2008	32	4.2	0.529	0.578	0.079	15	3	0.648	0.00538
	BN-08^+^	Mar 2009	40	3.9	0.568	0.609	0.068				
	BN-12^+^	Oct 2009	65	4.1	0.549	0.574	0.116	16	4	0.692	0.00466
	BN-24	Jun 2011	18	5.348	0.631	0.631	0.06	13	2	0.385	0.00244
Busiime	BU-0^∗+^	Mar 2008	39	3.5	0.476	0.485	0.046	17	1	0.000	0.0000
	BU-8^+^	Mar 2009	40	3.4	0.464	0.477	0.025				
	BU-12^+^	Oct 2009	40	3.4	0.464	0.489	0.037	19	1	0.0000	0.0000
Junda	JN-0^∗+^	Mar 2008	40	3.2	0.479	0.489	0.023	19	3	0.444	0.00731
	JN-13^+^	Jan 2010	18	3.1	0.460	0.485	0.065	18	1	0.000	0.00000
	JN-24	Jul 2011	3	3.091	0.407	0.333	−0.023	1			
Mukongoro	MK-0^∗+^	Mar 2008	40	2.9	0.487	0.460	−0.041	21	2	0.495	0.00093
	MK-08^+^	Mar 2009	24	3.0	0.455	0.431	−0.043				
	MK-12^+^	Nov 2009	22	3.1	0.418	0.445	0.068	21	2	0.467	0.00088
Masindi	MS-0^∗+^	Mar 2008	40	3.7	0.568	0.547	−0.025	18	2	0.467	0.001886
	MS-13^+^	Jan 2010	17	4.4	0.562	0.597	0.055	17	2	0.471	0.00964
	MS-25	Jul 2011	30	4.722	0.542	0.5533	0.039	15	2	0.419	0.00620
Okame	OK-0^∗+^	Mar 2008	39	3.3	0.452	0.507	0.081	17	3	0.471	0.0094
	OK-8^+^	Mar 2009	40	3.4	0.563	0.546	0.008	—	—	—	—
	OK-12^+^	Oct 2009	39	3.4	0.547	0.552	0.036	18	2	0.294	0.00055
Otuboi	OT-0^∗+^	Jul 2008	53	4.0	0.508	0.535	0.085	20	3	0.426	0.0122
	OT-11^+^	Nov 2009	40	3.7	0.514	0.540	0.076	20	4	0.537	0.00131

*N*: number of individuals analyzed, *A*_*R*_: allelic richness, *H*_*O*_: observed heterozygosity, *H*_*E*_: expected heterozygosity, *F*_IS_: Fisher's inbreeding coefficient; Hd: haplotype diversity, *π*: nucleotide diversity. Dashes indicate sites from which mtDNA was not collected, *indicates samples used in Beadell et al. (2010) [25], and ^+^indicates samples used in Echodu et al. 2011 [26].

**Table 2:** Results of AMOVA analyses on 15 microsatellite loci and mtDNA.

	D.f	Sum of squares	Variance component	% Variation	*P* value
Microsatellites					
Among sites	21	2215.937	0.99981	18.03	0.0000
Within sites	1118	5250.224	0.15046	2.71	0.0000
mtDNA					
Among sites		428.246	0.94483	64.01	0.05
Within sites		239.629	0.53133	35.99	0.05

D.f: degrees of freedom.

**Table 3:** Percent of pairwise comparisons of individuals that fell into each relatedness category (e.g., unrelated, half-sibling, or full sibling) as calculated in ML-Relate (Kalinowski, 2011 [27]). Comparisons were made among all individuals (All) and among individuals within each sampling site.

	All	BU	SA	OK	KIS	NB	TB	NAM	MGG	IGG	BN
Unrelated	85.8	82.5	94.2	83	92.8	85.3	89.9	92.5	85.3	84.7	83.9
Half-sibling	12	15.2	4.8	14.8	3.6	11.8	9	7.5	12.5	13.6	15
Full sibling	1	0.9	0	1.2	0	0.7	0.8	0	1.3	1	0.7
Parent-offspring	1.2	1.4	1	1	3.6	2.2	0.3	0	0.9	0.7	0.4

	BK	PT	BKD	MK	OT	OC	AM	MS	KF	KR	JN

Unrelated	82.3	82.8	84.3	77.6	84.1	92.1	84.8	82.1	84.3	89.2	83.2
Half-sibling	15.8	13.9	11.7	17.3	14.2	4.2	12.2	15.4	14.1	9.9	14.5
Full sibling	1	1.8	2	2.1	0.9	1.6	1.8	1.1	1.1	0.8	1.3
Parent-offspring	0.9	1.5	2	3	0.8	2.1	1.2	1.4	0.5	0.1	1

**Table 4:** Summary of first generation migrants by sampling locality. The first four columns report the sampling site, its symbol, and microsatellite and mtDNA haplogroups assignment. The last four columns list the total number of migrants detected in each site and how many of these migrants come from a site from the northern, the southern, or the western clusters.

Locality	Microsatellite cluster	mtDNA cluster	Total migrants	Northern	Southern	Western
BU	S	S	2	—	2	—
SA	S	S	2	—	2	—
OK	S	S	3	—	3	—
NB	S	S	4	—	4	—
TB	S	S	3	—	2	1
NAM	S	Mixed	6	—	4	2
MGG	S	S	4	—	4	—
IGG	S	S	4	—	4	—
BN	N	Mixed	8	5	1	2
BK	N	N	2	2	—	—
PT	N	N	1	1	—	—
BKD	N	N	0	—	—	—
MK	N	N	3	3	—	—
OT	N	N	3	3	—	—
OC	N	N	2	2	—	—
AM	N	N	2	2	—	—
KT	N	W	3	1	1	1
MS	W	W	5	—	3	2
KF	W	W	3	—	—	3
KR	W	W	2	—	1	1
JN	Mixed	Mixed	4	—	4	—
TR	S	S	3	—	3	—

**Table 5:** Sex-biased dispersal test results. Four different statistics were used: *F*_ST_, mean assignment indices (mAIc), the variance in assignment indices (vAIc), and mean pairwise relatedness (mPr). Results for both two- and one-sided tests are reported. Sex and samples sizes are reported in the first column. *P* values for each statistics are reported in the last row.

	*F*_ST_	mAIc	vAIc	mPr
Two-sided				
F (540)	0.1912	0.02368	22.5165	0.3106
M (581)	0.1915	0.02201	16.36478	0.3112
*P* value	0.9775	0.8642	0.8552	0.9503
One-sided				
F (540)	0.1913	0.02368	22.5165	0.3106
M (581)	0.1915	0.02201	16.36478	0.3112
*P* value	0.774	0.4392	0.5695	0.7847

## Appendix

1. Information on the study site, time of collection, and number of flies caught in each site.
2. Details of microsatellite markers used in the study.
3. Per locus estimates of *F*_IS_ at 18 microsatellite loci for each sampling locality.
4. Mitochondrial haplotype information, including frequencies observed across studied populations. New haplotypes recovered from this study are indicated by Richard Echodu and Mark Sistrom.
5. Results of an AMOVA testing for temporal, genetic structure in four populations of *G*. *fuscipes* sampled in 2008 and also in 2011.
6. *F*_ST_ values for temporal samples calculated on microsatellite data. Nonsignificant values are in bold.
7. Microsatellite-based *F*_ST_ values pairwise comparison between sampling localities of *G*. *f*. *fuscipes* in Uganda.
8. Microsatellite-based *F*_ST_ values for pairwise comparisons among the three populations detected using Bayesian clustering.
9. Details of all first generation migrants detected by GeneClass 2.0, using *L*_*h*_, *L*_*h*_/*L*_max⁡_, and STRUCTURE.
10. Comparison of mtDNA clade and microsatellite assignment for each individual where both data types were collected.
