# Supplementary figures and images for: Genetically Distinct Glossina fuscipes fuscipes Populations in the Lake Kyoga Region of Uganda and Its Relevance for Human African Trypanosomiasis
Source: Biomed Res Int. 2013 Oct 2;2013:614721. doi: 10.1155/2013/614721 (PMC3807537; doi:10.1155/2013/614721)

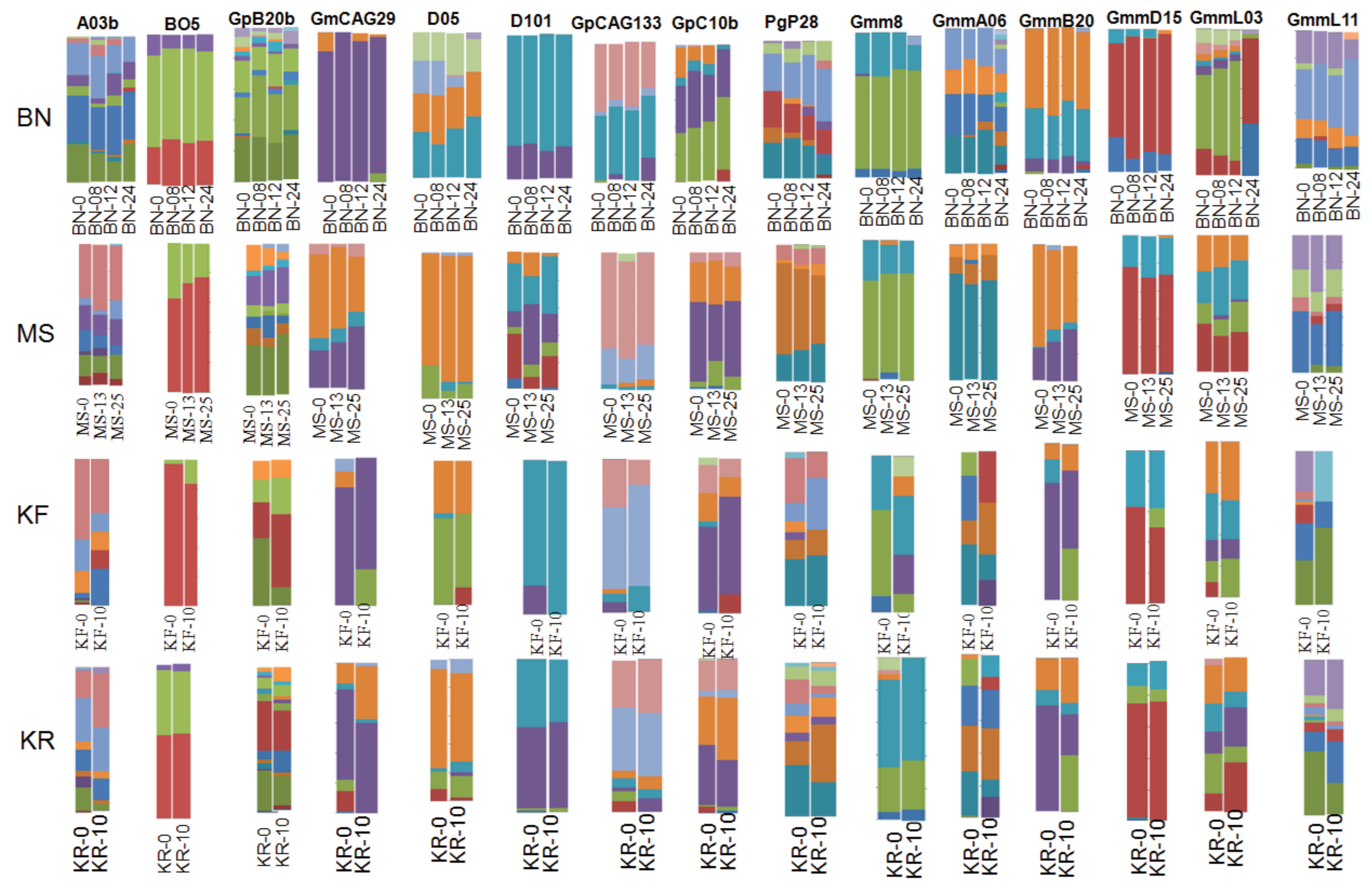

Supplement: Supplementary file 1 — Appendix 1: Information on the study site, time of collection and number of flies caught in each site. Appendix 2: Details of microsatellite markers used in the study. Appendix 3: Per locus estimates of FIS at 18 microsatellite loci for each sampling locality. Appendix 4: Mitochondrial haplotype information, including frequencies observed across studied populations. New haplotypes recovered from this study are indicated by ∗. Appendix 5: Results of an AMOVA testing for temporal genetic structure in four populations of G. fuscipes sampled in 2008 and also in 2011. Appendix 6: Fst values for temporal samples calculated on microsatellite data. Non-significant values are in bold. Appendix 7: Microsatellite-based FST values pairwise comparison between sampling localities of G. f. fuscipes in Uganda. Appendix 8: Microsatellite-based FST values for pairwise comparisons among the three populations detected using Bayesian clustering. Appendix 9: Details of all first generation migrants detected by Geneclass 2.0, using Lh, Lh/Lmax and STRUCTURE. Appendix 10: Comparison of mtDNA clade and microsatellite assignment for each individual where both data types were collected. Figure 1: Microsatellite allele frequencies observed in seven populations of G. f. 52 fuscipessampled at different time points. Numbers after location code indicate the time interval 53 (in generations) since the first sampling. [file 614721.f1.zip › 614721.f1/Supplementaryfigure1.pdf]
